# Supplementary material for: Hidden diversity: comparative functional morphology of humans and other species
Source: PeerJ. 2023 Apr 24;11:e15148. doi: 10.7717/peerj.15148 (PMC10135406; doi:10.7717/peerj.15148)
Supplement: Supplemental Information 1 [file peerj-11-15148-s001.docx]

# A Diversity of Guts

### Cadaver Dissection Information and Measurements for Comparative Analysis of Morphological Variation

**Program Information:**

- Institution:
- Program of Study (all lab partners):
- Course:
- Course Instructor:
- Cadaver Identification number (see index card):
- Table number (T on index card):
- Cadaver sex (see index card):
- Cadaver age (see index card):
- Cadaver cause of death (COD) (see index card):

**All measurements recorded in centimeters (cm)**

**Stomach:**

1. **Fundus to pylorus** **(ruler tape)**: Measure the linear length from the uppermost portion of the fundus to the center of the pyloric sphincter.
2. **Width of the pylorus (calipers)**: Measure the cross-sectional width of the pyloric region at the pyloric sphincter.
3. **Lesser curvature of the stomach (string and ruler tape)**: Using a segment of string, place one end at the cardioesophageal sphincter and follow the inner curvature to the center of the pyloric sphincter. Measure the length of string.
4. **Greater curvature of the stomach (string and ruler tape)**: Using a segment of string, place one end at the cardioesophageal sphincter and follow the outer curvature to the center of the pyloric sphincter. Measure the length of string.

**Liver:**

1. **Vertical length (ruler tape)**: Measure the liver from the inferior extent of the right lobe to the superior extent of the right lobe (this should be the most superior point on the right lobe).
2. **Horizontal length (ruler tape)**: Measure the liver from the supero-lateral aspect of the right lobe to the most medial aspect of the left lobe.
3. **Maximum length (ruler tape)**: Measure the liver from the most inferior aspect of the right lobe to the most medial aspect of the left lobe. This is a linear distance between two points not following the organ.
4. **Liver volume (ruler tape)**: Take the following measurements to calculate liver volume using the equation *V=πr^2^(h/3)*.
   1. **Height of liver (ruler tape)**: Find the hilum of the liver. Measure the linear distance from the hilum to the apex of the liver. This value represents (*h*) in the formula above.
   2. **Radius of liver**: Using the #7 (Maximum Length of Liver). This value is 2*radius (*r*) in the formula above and will need to be divided by 2 to calculate volume.
   3. **Liver volume:** $V= \pi r^{2}\frac{h}{3}$

**Gallbladder:**

1. **Presence/Absence**
2. **Maximum length (calipers)**: Measure the gallbladder from the fundus to the start of the cystic duct.

**Pancreas:**

1. **Maximum length (ruler tape and string)**: Measure the distance between the head and tail along the long axis of the pancreas.

**Spleen:**

1. **Presence/Absence**
2. **Maximum length (calipers)**: Measure the spleen from the superior to inferior poles.
3. **Circumference (ruler tape)**: Measure the maximum circumference around the spleen. This is typically at the hilum, you can puncture a hole in the membrane to run the tape through.

**Small Intestine:**

1. **Duodenum:**
   1. **Length (string and ruler tape)**: Measure the distance from the pyloric sphincter to the duodeno-jejunal flexure (aka the ligament of Treitz) using the string. Measure the length of string.
   2. **Width (calipers)**: Measure the width of the duodenum just distal to the pyloric sphincter.
2. **Jejunum and Ileum:**
   1. **Length (string and ruler tape):** Cut a 500 cm length of string to start. Using the string follow the jejunum and ileum from the duodeno-jejunal flexure to the ileocecal valve. Measure the length of string.
   2. **Width of jejunum (calipers)**: Measure the width of the jejunum just distal to the duodeno-jejunal flexure.
   3. **Width of ileum (calipers)**: Measure the width of the ileum just proximal to the ileo-cecal junction.

**Cecum:**

1. **Maximum length (ruler tape)**: Measure the cecum from its free base to the region attached by peritoneum (along the superior to inferior axis).
2. **Width (calipers)**: Measure the point of maximum width of the cecum.

**Appendix:**

1. **Presence/Absence**
2. **Maximum length (ruler tape)**: Measure the appendix along its long axis from the base of the cecum to the tail.

**Colon: Straighten each region of the colon as much as possible.**

1. **Length of ascending colon (string and ruler tape):** Measure from the retroperitoneal portion at the distal cecum to the right colic flexure.
2. **Width of ascending colon (calipers):** Measure the width just distal to the transition from the cecum to the ascending colon. Note: Avoid overly compacted or distended regions.
3. **Length of transverse colon (string and ruler tape):** Measure the length from the right colic flexure to the left colic flexure.
4. **Width of transverse colon (calipers):** Measure the width just distal to the right colic flexure. Note: Avoid overly compacted or distended regions.
5. **Length of descending colon (string and ruler tape):** Measure the length from the left colic flexure to the start of the sigmoid colon (distal portion free from peritoneum).
6. **Width of descending colon (calipers):** Measure the width just distal to the left colic flexure. Note: Avoid overly compacted or distended regions.
7. **Length of sigmoid colon (string and ruler tape)**: Using the string, follow the sigmoid colon from the portion at the edge of the peritoneum to the rectal transition. Measure the string.

**Length of rectum (ruler tape):** Measure from the distal sigmoid colon to the just above the anal sphincter. Note: Attempt to get a maximum length measurement when possible.
