# Supplementary material for: Hidden diversity: comparative functional morphology of humans and other species
Source: PeerJ. 2023 Apr 24;11:e15148. doi: 10.7717/peerj.15148 (PMC10135406; doi:10.7717/peerj.15148)
Supplement: Supplemental Information 2 [file peerj-11-15148-s002.docx]

A Diversity of Guts

Vertebrate Dissection Information and Measurements for Comparative Analysis of Morphological Variation

## Materials

| Gloves | String |
| --- | --- |
| Safety Glasses | Dissection Scissors |
| Ruler Tape | Scalpel |
| Ruler (cm) | Scale (if available) |
| Dissecting pins | Dissecting pan and pad |

## Measurement Definitions

**The following measurements are taken prior to dissection. All measurements should be collected in centimeters (cm) except for body mass:**

1. **Body Length**
   1. **Bregma to Ischium** (ruler tape) - Measure the dissection specimen from landmarks bregma on the anterior portion of the animal to ischium on the posterior of the animal. *Using the ruler tape measure from the central most point on the head to the bony protrusions of the pelvis along the back. Note: Ensure the animal is as straight as possible before measuring.*
   2. **Tip of nose to tip of tail** (ruler tape)- Measure the dissection specimen from the tip of the nose (*most anterior point on the head*) to the tip of the tail (*most posterior point of the body*).

**As you begin the dissection of your animal, it is important to remember that the digestive tract is a single tube that runs from mouth to anus. In order to remove the digestive tract in entirety from your animal you will only make two simple cuts, one at the top of the esophagus and one at the anus. As you begin to tease out and lift the digestive tract out of your animal you will discover that there is tissue holding the digestive system in place around the thoracic cavity as well the abdominal cavity (peritoneum and mesentery). It is important that you work slowly and carefully with a scalpel to cut adhering tissues as you work to remove the digestive tract. Once the digestive tract has been removed, and teased apart, you can begin your measurement collection.**

1. **Length of Esophagus** (ruler)- At the most anterior location of the digestive tract measure the distance in centimeters between the upper esophageal sphincter and the lower esophageal sphincter (*opening of the stomach*).
2. **Length of Stomach** (string and ruler)- Directly posterior to the esophagus, measure the distance in centimeters between the lower esophageal sphincter and the pyloric sphincter (*Note: use string to measure the maximum length including the curve of the stomach, then measure the length of the string*)
3. **Length of Small Intestine** (string and ruler) - Directly posterior to the stomach, measure the distance in centimeters from the pyloric sphincter at the junction of the stomach and the duodenum to the ileocecal junction between the ileum and the cecum. (*Note: some small intestines will be too large to measure directly. Using string and dissecting pins follow the small intestine and then measure the string length.*)
4. **Length of Cecum (if present)** (ruler)- Locate the cecum at the junction between the small and large intestines. Measure the total length of the cecum from the ileocecal junction to the cecocolic junction with the large intestine.
5. **Length of Colon** (ruler) - Measure the length of the large intestine in centimeters from the cecocolic junction to the anorectal junction.
