# Supplementary material for: Hidden diversity: comparative functional morphology of humans and other species
Source: PeerJ. 2023 Apr 24;11:e15148. doi: 10.7717/peerj.15148 (PMC10135406; doi:10.7717/peerj.15148)
Supplement: Supplemental Information 3 [file peerj-11-15148-s003.docx]

**Table S1. Comparison of means ± standard deviation (in cm) from the present study to published literature, where available, for rat (*Rattus norvegicus*), frog (*Lithobates catesbeianus*), and fetal pig (*Sus scrofa*) specimens.**

|  | **n** | **Length of Small Intestine (cm)** | **Length of Cecum (cm)** | **Length of Colon (cm)** |
| --- | --- | --- | --- | --- |
| **Rat** | | | | |
| Present Study | 10 | 91.26 ± 0.94 | 9.215 ± 0.202 | 14.41 ± 0.140 |
| Permezel & Webling (1971) | 25 | 67.1 | 2.5 ± 0.1 | 10.2 ± 0.2 |
| Fisher & Parsons (1950) | 50 | 111.2 ± 1.159 | NR**^1^** | NR**^1^** |
| **Frog** | | | | |
| Present Study | 10 | 19.08 ± 0.13 | -- | 3.49 ± 0.13 |
| Toloza & Diamond (1990) | 20 | 22.2 ± 1.1 | -- | 3.3 ± 0.4 |
| **Fetal Pig** | | | | |
| Present Study^2^ | 10 | 274.705 ± 1.902 | 4.13 ± 0.02 | 51.67 ± 0.38 |
| Wang et al., (2005)^2^ | 5 | 244.04 ± 24.31 | NR**^1^** | 69.84 ± 3.55 |
| Miller & Ullrey (1987)^3^ | NR^1^ | 380 | NR**^1^** | 80 |

^1^NR = not reported.

^2^age: newborn

^3^age: 1 day
