# Supplementary material for: Hidden diversity: comparative functional morphology of humans and other species
Source: PeerJ. 2023 Apr 24;11:e15148. doi: 10.7717/peerj.15148 (PMC10135406; doi:10.7717/peerj.15148)
Supplement: Supplemental Information 4 — Standard deviation values were only available for Hirsch (1955) and the present study. Sample sizes were not reported in gross anatomy texts. [file peerj-11-15148-s004.docx]

**Table S2. Comparison of means ± standard deviations (in cm) from the present study to published literature and most-recommended gross anatomy texts (denoted with an asterisk) for humans (*Homo sapiens*).** Standard deviation values were only available for Hirsch (1955) and the present study. Sample sizes were not reported in gross anatomy texts.

|  | **n**^1^ | **Length of Small Intestine (cm)** | **Length of Appendix (cm)** | **Length of Colon (cm)** |
| --- | --- | --- | --- | --- |
| **Present Study** | 37 | 304.955 ± 84.331 | 8.127 ± 2.484 | 122.119 ± 30.755 |
| **Treves (1885)** | 100 | 698.5**^2^** | NR**^1^** | 139.7**^2^** |
| **Dreike (1894)** | 40 | 601.7 | NR**^1^** | 150.4 |
| **Bloch (1904)** | 20 | 641.5 | NR**^1^** | 143.93 |
| **Bryant (1924)** | 44 | 624.84 | NR**^1^** | 152.4 |
| **Blankenhorn, Hirsch & Ahrens (1955)** | 10 | 284.98 ± 43.2**^3^** | NR**^1^** | 133 ± 10.5 |
| **Underhill (1955)** | 100 | 612.95 | NR**^1^** | 196.29 |
| **Standring, Borley & Gray (2008)*** | NR**^1^** | 609.6 (Duodenum ~25.4) | NR**^1^** | 152.4 |
| **Netter (2014)*** | NR**^1^** | 600 (Duodenum ~25; Jejunum ~250; Ileum ~350) | 7 - 10 | 150 |
| **Tubbs, Shoja, & Loukas (2016)*** | NR**^1^** | 600 (range 350-700) | 6 - 10 | 150-200 |

^1^NR = not reported.

^2^Male and female averages.

^3^Small intestine lengths only available for 5 individuals.
