# Supplementary material for: Hidden diversity: comparative functional morphology of humans and other species
Source: PeerJ. 2023 Apr 24;11:e15148. doi: 10.7717/peerj.15148 (PMC10135406; doi:10.7717/peerj.15148)
Supplement: Supplemental Information 5 [file peerj-11-15148-s005.docx]

**Table S3. Pearson product-moment correlation for non-human animal species, for each of the measurements examined.**

| **Species** | **Rat** | **Pig** | **Frog** |
| --- | --- | --- | --- |
| **Small intestine vs cecum length** | Corr = 0.4593  p = 0.1818 | Corr = 0.0373  p = 0.9184 | NA |
| **Small intestine vs colon length** | Corr = 0.4644  p = 0.1763 | Corr = 0.3178  p = 0.3709 | Corr = -0.1095  p = 0.7633 |
| **Cecum vs**  **colon length** | Corr = 0.4906  p = 0.1499 | Corr = 0.03799  p = 0.917 | NA |
