# Supplementary material for: Hidden diversity: comparative functional morphology of humans and other species
Source: PeerJ. 2023 Apr 24;11:e15148. doi: 10.7717/peerj.15148 (PMC10135406; doi:10.7717/peerj.15148)
Supplement: Supplemental Information 6 — All lengths are provided in cm; liver volume is in cm 3. Significantly different values are in bold (α = 0.05). [file peerj-11-15148-s006.docx]

**Table S4. Test statistics, degrees of freedom, p-value, and Pearson correlations for the reported results in Table 2.** Significant results are in bold (α = 0.05).

| **Correlation variables** | **df** | **t-statistic** | **p-value** | **Correlation** |
| --- | --- | --- | --- | --- |
| **Liver volume-Length of gall bladder (max)** | 27 | -0.3565 | 0.7242 | -0.0685 |
| **Liver volume-Length of cecum** | 39 | 1.8186 | 0.0767 | 0.2796 |
| **Liver volume-Length of small intestine** | 30 | 1.0595 | 0.2978 | 0.1899 |
| **Liver volume-Length of appendix** | 28 | 2.5278 | **0.0174** | 0.4311 |
| **Liver volume-Length of colon** | 19 | 2.0991 | **0.0494** | 0.4339 |
| **Length of gallbladder-Length of cecum** | 28 | -0.8124 | 0.4234 | -0.1517 |
| **Length of gallbladder-Length of small intestine** | 22 | -0.1742 | 0.4826 | -0.1505 |
| **Length of gallbladder-Length of appendix** | 23 | 0.9744 | 0.3400 | 0.1991 |
| **Length of gallbladder-Length of colon** | 15 | -0.3093 | 0.7613 | -0.0796 |
| **Length of cecum-Length of small intestine** | 31 | 1.4234 | 0.1646 | 0.2477 |
| **Length of cecum-Length of appendix** | 31 | 0.9484 | 0.3502 | 0.1679 |
| **Length of cecum-Length of colon** | 21 | 1.108 | 0.2804 | 0.2350 |
| **Length of small intestine-Length of appendix** | 22 | 1.8657 | 0.0755 | 0.3696 |
| **Length of small intestine-Length of colon** | 17 | 2.1699 | **0.0445** | 0.4657 |
| **Length of appendix-Length of colon** | 18 | 0.7899 | 0.4399 | 0.1830 |
