# Supplementary material for: Hidden diversity: comparative functional morphology of humans and other species
Source: PeerJ. 2023 Apr 24;11:e15148. doi: 10.7717/peerj.15148 (PMC10135406; doi:10.7717/peerj.15148)
Supplement: Supplemental Information 7 — Significant results are in bold (α = 0.05). [file peerj-11-15148-s007.docx]

**Table S5. Summary statistics for the GI organs across the human cadaver sample, by sex.** All lengths are provided in cm; liver volume is in cm^3^. Significantly different values are in bold (α = 0.05).

| Measurements | n | | Mean | | Min | | Max | | Std Dev | | C_v_ | |
| --- | --- | --- | --- | --- | --- | --- | --- | --- | --- | --- | --- | --- |
| sex | M | F | M | F | M | F | M | F | M | F | M | F |
| Body length | 23 | 21 | 175.9 | 159.7 | 156.2 | 149.0 | 184.0 | 173.0 | 6.65 | 6.51 | 3.78 | 4.08 |
| Liver volume (cm^3^) | 22 | 19 | 1227.57 | 1113.55 | 377.38 | 384.85 | 2682.22 | 2872.53 | 643.16 | 651.30 | 52.385 | 58.489 |
| Length of Gallbladder (maximum) | 17 | 13 | 8.62 | 9.15 | 6.4 | 5.5 | 11.2 | 12.5 | 1.43 | 1.92 | 16.57 | 20.93 |
| Length of Small intestine | 17 | 16 | **404.15** | **434.88** | 214.9 | 291.4 | 519.2 | 610.2 | 74.22 | 93.68 | 18.37 | 21.54 |
| Length of Duodenum | 17 | 16 | 29.27 | 25.00 | 18.9 | 17.1 | 46.3 | 46.2 | 7.56 | 6.72 | 25.82 | 26.87 |
| Length of Jejuno-ileum | 23 | 20 | 386.97 | 396.83 | 193.50 | 266.50 | 501.40 | 592.00 | 70.75 | 88.57 | 18.28 | 22.32 |
| Length of Cecum | 24 | 20 | 13.05 | 12.23 | 6.61 | 6.5 | 25.8 | 21.7 | 4.64 | 3.72 | 35.52 | 30.41 |
| Length of Appendix | 19 | 14 | 6.71 | 7.18 | 1.4 | 3.3 | 12 | 12.7 | 2.68 | 2.26 | 39.91 | 31.53 |
| Length of Colon | 12 | 11 | 136.73 | 132.55 | 80.9 | 100.8 | 199 | 176.6 | 36.90 | 23.93 | 26.99 | 18.06 |
| Total intestinal length | 12 | 11 | **451.13** | **524.13** | 80.9 | 137.5 | 718.2 | 758.5 | 214.54 | 168.26 | 47.56 | 32.10 |
